# Antimicrobial Stewardship and Dose Adjustment of Restricted Antimicrobial Drugs in Hospital Setting

**DOI:** 10.3390/pharmacy11020068

**Published:** 2023-04-02

**Authors:** Iva Vlak, Ivana Samardžić, Ivana Marinović, Nikolina Bušić, Vesna Bačić Vrca

**Affiliations:** 1 Department of Clinical Pharmacy, University Hospital Dubrava, 10000 Zagreb, Croatia; 2 Department of Hospital Infections and Antimicrobial Stewardship, University Hospital Dubrava, 10000 Zagreb, Croatia; 3 Faculty of Pharmacy and Biochemistry, 10000 Zagreb, Croatia

**Keywords:** Antimicrobial stewardship, restricted antimicrobial drugs, dose adjustment, adverse drug reactions

## Abstract

Antimicrobial consumption is increasing. In order to maximize the effectiveness of antimicrobial stewardship and provide safe and optimal use of restricted antimicrobial drugs, renal dosing should be evaluated. The aim of this study was to determine the prevalence of restricted antimicrobial drugs that required dose adjustment according to renal function. A retrospective, consecutive study was conducted at University Hospital Dubrava. This study analyzed requests for restricted antimicrobial drugs (n = 2890) during a 3-month period. Requests for antimicrobial agents were evaluated by the antimicrobial therapy management team (A-team). This study included 412 restricted antimicrobial drug requests requiring dose adjustment, of which 39.1% did not have an adjusted dose. Meropenem, Ciprofloxacin, Piperacillin/Tazobactam, Vancomycin, Colistin and the antimycotic Fluconazole were the most frequent restricted antimicrobial drugs that required dose adjustment according to impaired renal function. The results of this research highlight the importance of the A-team in the optimization of restricted antimicrobial therapy. Non-adjusted doses of restricted antimicrobial drugs increase the possibility of adverse drug reactions and therefore jeopardize pharmacotherapy outcomes and patient safety.

## 1. Introduction

The World Health Organization (WHO) has identified antimicrobial resistance (AMR) as one of the ten greatest public health threats to humanity. Uncontrolled and excessive use of antimicrobial agents leads to the development of resistant pathogens, encouraging bacterial adaptation [1,2,3]. The European Center for Disease Prevention and Control (ECDC) estimates that 33,000 people die annually due to antimicrobial resistance, and the annual cost of treating such infections is 1.1 billion euros in the European Union/EEA. It is predicted that by 2030, the resistance to second-line antimicrobial therapy will be 72% higher than it was in 2005, while resistance to last-line antimicrobial therapy will be more than double [4]. In Croatia, in 2021, systemic anti-infective drug use was ranked as the 9th highest in total drug consumption (18.21 defined daily doses per 1000 inhabitants per day) [5]. The percentage of antimicrobial consumption is increasing [6,7,8]. 

Antimicrobial stewardship (AMS) is a coherent set of actions which measures, optimizes and improves antimicrobial drug use [9]. The goals of AMS are to change inappropriate prescribing and excessive use of antimicrobial drugs in order to preserve antibiotic effectiveness and limit further spread of resistant microorganisms. AMS actions comprise evaluation of antimicrobial therapy prescriptions, providing feedback to drug prescribers, setting antimicrobial therapy guidelines and educating prescribing physicians [10]. AMS actions improve healthcare, increase patient safety and reduce inpatient therapy costs [11]. Nowadays, the importance of AMS is of particular interest since there is high consumption of existing antibiotics, and few new ones are entering the pharmaceutical market [12,13,14]. Effective antimicrobial agents are necessary for the success and further development of modern medicine [1]. Creating a list of restricted antimicrobial drugs is a part of the actions aimed to preserve existing antimicrobial drugs [15]. Therefore, optimized antimicrobial drug use is of special interest [16,17,18].

AMS is conducted by the hospital’s delegated antimicrobial therapy management team (A-team). The A-team must include infectious disease specialists and a clinical pharmacist as core members along with clinicians, clinical microbiologists, hospital epidemiologists and other specialists [19]. It is important to outline that the A-team requires a multidisciplinary team approach. Program personnel should be included as active members in the hospital infection control and pharmacy and therapeutics committees. Key to implementing an effective program is a proactive strategy that includes prospective audit with direct intervention and feedback to the provider for antimicrobial drug use [19].

Previously published studies on the effectiveness of A-teams have mostly published results regarding consumption and cost reductions [20]. There are few published studies that deal with issues regarding other determinants of AMS that are part of patient safety [21]. In Europe, an average of 4.6% of all hospital admissions are due to adverse drug reactions (ADRs). Further, the incidence of ADRs during hospitalization in Europe is 17% on average [22]. A recent report showed that 20% of admitted patients treated by antimicrobial therapy developed an antibiotic-associated adverse drug event (ADE) [23]. Hospitalized patients, who are generally older with multimorbidity and polytherapy and receive parenteral therapy in higher doses than they would by using oral regimens, are more vulnerable to ADEs compared to outpatients [24,25,26]. Dose optimization is a part of AMS and an important segment in providing patient safety [19]. A prerequisite for minimizing the risk of ADRs is dose adjustment, especially in patients with impaired renal function. 

Chronic kidney disease (CKD) is a major global health problem which affects 11 to 13% of the general population, which is even higher in elderly and hospitalized patients [27,28]. An increased presence of CKD is associated with drug toxicity. Drug-induced toxicity and ADRs are more likely to occur in patients who do not have an adjusted drug dose. Avoidance of ADRs is essential in hospitalized patients who are usually of poor general condition showing multimorbidity and various clinical complications [24]. In order to maximize the effectiveness of AMS and provide safe and optimal use of restricted antimicrobial drugs, renal dosing should be evaluated.

The aim of this study was to determine the prevalence of restricted antimicrobial drugs requiring dose adjustment according to renal function as a part of AMS. 

## 2. Materials and Methods

A retrospective, consecutive study was conducted at University Hospital Dubrava (UHD), in Croatia during a 3-month period. UHD is a tertiary care health institution with 600 beds, whose emergency medical service covers a population of about 350,000 inhabitants. The A-team of the UHD comprises clinicians of different specialties, a clinical microbiologist, an infectious disease specialist and a clinical pharmacist. They are active members of the therapeutic committee, the committee of hospital infections, and the working group for antibiotic management of commission for the quality of healthcare. Our study was exempt from providing informed consent since the data were anonymized for antimicrobial stewardship activities.

This study analyzed requests for restricted antimicrobial drugs from all hospital departments, except for the Department for Cardiac and Transplantation Surgery due to the specific way of issuing/dispensing drugs through the Unit Dose Drug Distribution System. In UHD, in order to issue a restricted antimicrobial drug, a special document that contains the following categories is to be filled:

- name of department/clinic;
- patient information including age and weight;
- clinical data: main diagnosis for use of restricted antimicrobials, other diagnoses, renal function (normal or impaired), type of infection (out-of-hospital or in-hospital), type of antibiotic therapy (empirical, targeted, prophylaxis or continuation of already approved therapy), previous antimicrobial therapy, microbiological sample, isolated causative agent and sensitivity to antimicrobials (in case of targeted therapy);
- name of the required antimicrobial drug and daily dosage;
- special observations and prescriber’s explanation for the required antimicrobial;
- section for clinical pharmacologist’s opinion/commentary;
- section for infectologist’s opinion/comment;
- section for clinical pharmacist’s opinion/commentary.

A restricted antimicrobial drug is issued in quantity for a three-day therapy application, followed by a re-evaluation of antimicrobial therapy and a renewed request form. At UHD, a physician fills out and signs a request which is then transferred to the Central Hospital Pharmacy where the A-team reviews and approves the issuance and application of the requested restricted antimicrobial drugs. The clinical pharmacist evaluates if the stated daily dosage should be adjusted according to the patient’s renal function. 

Data for renal function were documented upon each request. The Kidney Disease: Improving Global Outcomes (KDIGO) classification of renal impairment was used, which included categories G1–G5 based on the estimated glomerular filtration rate (eGFR) values [29]. The eGFR, calculated from serum creatinine by using the CKD-epidemiology collaboration (CKD-EPI) formula, was taken from the laboratory report [30]. The UHD clinical laboratory has implemented the CKD-epidemiology collaboration (CKD-EPI) equations according to the national recommendations for laboratory testing [31]. Patients pertaining to stages 3a, 3b, 4, and 5 (eGFR < 60 mL/min/1.73 m^2^) were considered to have renal impairment. The summary of Product Characteristics (SmPC) or the UptoDate database were used to determine the adjustment of the antimicrobial dose. 

A clinical pharmacist documents pharmacotherapy problems upon the request under the section for clinical pharmacist’s opinion/commentary and presents them to the A-team. In order to issue a restricted antimicrobial drug, it was necessary to reach a consensus between the A-team members. All inadequate dosages were corrected by the A-team and then referred to prescribers. All A-team dose adjustment interventions were recorded.

Python 3.7 and Excel Office programs were used for data management and analyses. Descriptive statistics were used to describe the characteristics of the study population and results.

## 3. Results

A total of 2890 requests for 913 patients were included in this work. The proportion of male patients was significantly higher and the average age of the patients was 67.5 years (17–97). The share of elderly patients (≥65 years) was 64.6%. The mean number of requests for restricted antimicrobial drugs was 3.17 per patient. The most common isolated pathogens were *A. baumannii, K. pneumoniae* and *P. aeruginosa*. A total of 35 departments required restricted antimicrobials in the observed period. Patient characteristics are shown in Table 1.

**Figure 1:**
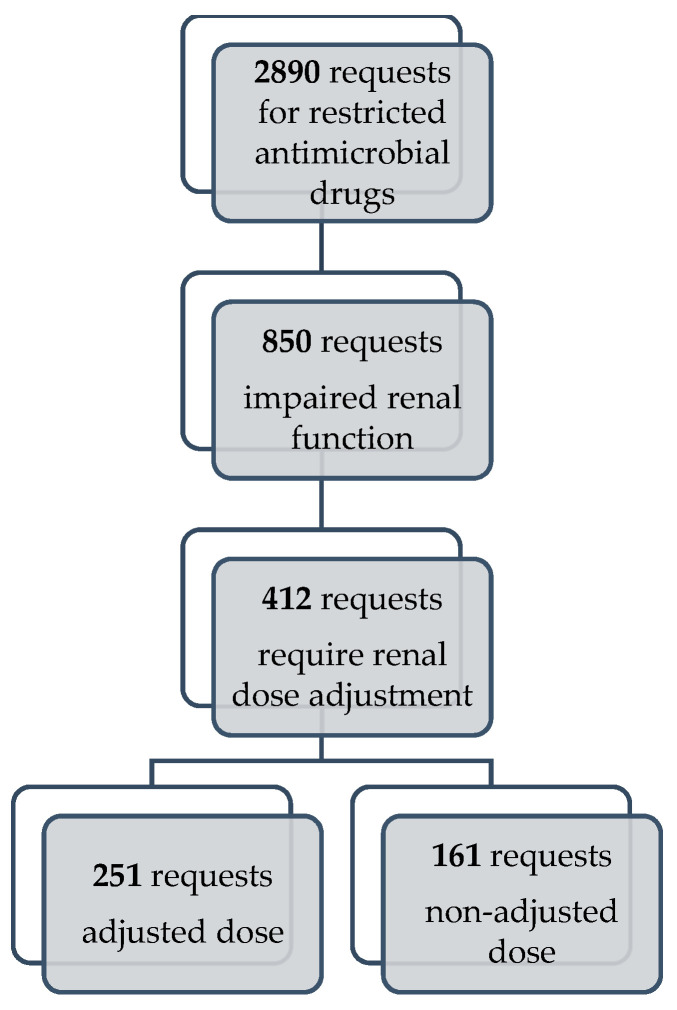
Total number of requests for restricted antimicrobial drugs, total number of requests for patients with renal impairment, total number of requests which required dose adjustment, and number of requests with non-adjusted antimicrobial dose.

Figure 1 represents the number of requests for restricted antimicrobial drugs analyzed in this study. Among 2890 requests, impaired renal function (eGFR < 60 mL/min/1.73 m^2^) was verified in 29.5% requests (n = 850). Overall, we identified 412 requests with restricted antimicrobial drugs requiring dose adjustment according to impaired renal function. Out of these 412 requests, 161 did not have an adjusted dose (39.1%), which corresponds to 5.6% of all analyzed requests for restricted antimicrobial drugs.

Table 2 represents restricted antimicrobial drugs which require renal dose adjustment and the number of requests. A total of 31 different restricted antimicrobial drugs were recorded in analyzed requests, of which 20 required renal dose adjustment and were stated in 91% of the requests (n = 2630). The most frequent requests for restricted antimicrobial drugs included Meropenem, Ciprofloxacin, Ceftriaxone, Vancomycin and Fluconazole. The restricted antimicrobial drugs that most frequently required renal dose adjustment were Meropenem, Ciprofloxacin, Piperacillin/Tazobactam, Vancomycin, Colistin and Fluconazole. The ratio of requests with an unadjusted dose of restricted antimicrobial drug to the number of requests that required renal dose adjustment is also shown in Table 2. 

There were 11 different restricted antimicrobial drugs which do not require dose adjustment in patients with renal function impairment, stated on 260 requests (Table 3). Linezolid was the most common restricted antimicrobial drug in this group.

**Figure 2:**
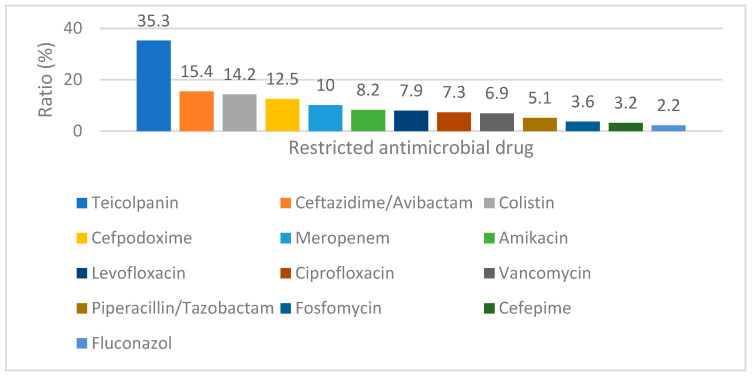
Ratio (%) between the number of requests with a non-adjusted dose and total number of requests for specific restricted antimicrobial drugs.

Figure 2 shows the ratio between the number of requests with a non-adjusted dose and the total number of requests for specific restricted antimicrobial drugs. Teicoplanin, ceftazidime/avibactam and colistin were identified as the restricted antimicrobial drugs with the highest ratio.

To summarize the results, a total of 2890 requests were analyzed in the observed period. Impaired renal function (eGFR < 60 mL/min/1.73 m^2^) was verified in 29.5% (n = 850) of requests. There were 412 (14.2%) requests for restricted antimicrobial drugs requiring renal dose adjustment of which 39.1% (n = 161) did not have a properly adjusted dose. For these 161 requests, the A-team adjusted the dose based on an agreement and these dose corrections were later referred to a prescriber. Meropenem, Ciprofloxacin, Piperacillin/Tazobactam, Vancomycin, Colistin and the antimycotic Fluconazole were the drugs that most often required renal dose adjustment.

## 4. Discussion

According to The Institute of Medicine’s (IOM) first Quality Chasm report, To Err is Human: Building a Safer Health System, antibiotics are among the most common classes of drugs associated with adverse outcomes [32]. Decreased renal function (G2–G5) was determined in half of all analyzed requests. Over 50% of the patients were elderly patients (≥65 years old). With aging, the level of renal function decreases [28]. With aging and renal impairment, the incidence of ADRs is up to 10 times higher [33]. ADRs prolong hospital stay [34]. Understanding ADRs is a prerequisite for achieving rational drug use. 

Meropenem was identified as the most frequently prescribed restricted antimicrobial drug. Meropenem shows a broad spectrum of activities against Gram-negative bacteria, including strains that produce Extended Spectrum Beta-Lactamase (ESBL), anaerobes and some Gram-positive bacteria. In total, 125 Meropenem requests (23.1% of all received Meropenem requests) required renal dose adjustment, of which 43.2% did not have a dose adjusted according to renal function. Meropenem dose accumulation increases the risk of ADRs such as seizures, delirium and continuous epileptiform outbursts. According to the FDA’s drug approval documentation, the following list ranks in the incidence of seizures caused by Carbapenems in descending order: Meropenem (0.7%), Ertapenem (0.5%) and Imipenem (0.4%). Risk factors that may contribute to development of epileptic seizures are high-dose therapy (>25 mg/kg), renal impairment and preexisting neurologic disorders [35]. 

Ciprofloxacin was the second most commonly used restricted antimicrobial drug. Among 121 Ciprofloxacin requests for patients with renal impairment (which is 26.6% of all received Ciprofloxacin requests), 27.3% had an unadjusted dose. The FDA and European Medicines Agency’s (EMA’s) Pharmacovigilance Risk Assessment Committee (PRAC) recommended that Fluoroquinolone antimicrobial therapy use should be restricted. Fluoroquinolone antibiotics can cause long-lasting, disabling and potentially permanent ADRs targeting tendons, muscles, joints and the nervous system. Tendon swelling and injury may occur 2 days after the therapy is started and up to several months after treatment discontinuation. Fluoroquinolones should generally be avoided in patients who previously had serious ADRs with a fluoroquinolone antibiotic and should be used under increased surveillance in elderly patients with impaired renal function, patients with concomitant corticosteroid use and transplant patients [36]. Furthermore, a recent meta-analysis reports a positive correlation between fluoroquinolone use and aortic aneurysm or dissection development, especially in patients with prolonged fluoroquinolone treatment and in the elderly [37]. 

In 39 Piperacillin/Tazobactam requests that required renal dose adjustment, 25.6% requests did not have an adjusted dose. High doses of Piperacillin/Tazobactam are particularly associated with neurotoxicity that may primarily manifest as consciousness disorders, hyperreflexia, myoclonism, and convulsions. The toxic effect on the central nervous system (CNS) is generally a less recognized ADR of antibiotics. It is well known that Penicillins cause a wide spectrum of neurotoxic manifestations [38,39]. Risk factors associated with neurotoxicity are age ≥65 years, decreased renal function, medical history of CNS disorders and/or damaged blood–brain barrier, increased body mass index, and co-administration of other neurotoxic or nephrotoxic medications [40]. 

Colistin and Vancomycin were among the top five restricted antimicrobial drugs with the highest number of requests requiring renal dose adjustment. Renal impairment increases the risk of ADRs associated with parenteral Vancomycin administration. Vancomycin is a nephrotoxic agent. The duration of vancomycin application and concomitant use of other nephrotoxic drugs increase the risk of nephrotoxicity. Oral use of Vancomycin is indicated for *Clostridium difficile* enterocolitis. Systemic absorption of oral Vancomycin from the digestive tract is negligible. However, with severe inflammation of the intestinal mucosa, especially in combination with renal insufficiency, the occurrence of ADRs associated with parenteral administration of Vancomycin is possible. Renal impairment and an unadjusted dose are potential risk factors for Vancomycin ototoxicity as well [41]. However, the most common ADRs associated with parenteral use of Vancomycin are phlebitis, pseudoallergic reactions and red neck syndrome. It is also important to highlight severe cutaneous adverse reactions of Vancomycin: Stevens–Johnson syndrome (SJS), toxic epidermal necrolysis (TEN), drug rash with eosinophilia and systemic symptoms (DRESS), and acute generalized exanthematous pustulosis (AGEP) [42]. 

Nephrotoxicity and neurotoxicity are the most common reported ADRs caused by Colistin therapy [43]. The likelihood of Colistin-mediated ADRs correlates with age, the patient’s clinical condition and renal function. ADRs associated with impaired renal function were recorded due to higher doses in patients with normal renal function and in patients with impaired renal function with unadjusted doses [44]. The risk of nephrotoxicity further increases with the parallel administration of other nephrotoxic drugs. A meta-analysis published in 2021 showed a Colistin-associated nephrotoxicity rate of 36.2% [45]. 

Antimycotics present a smaller part of the requests for restricted antimicrobial therapy. The analyzed period showed requests for 7 different antimycotics, representing 8.8% of the total number of requests. Fluconazole was the most frequently prescribed restricted antimycotic drug. In total, 230 requests were prescribed for Fluconazole, of which 21 required renal dose adjustment. In 21 Fluconazole requests which required renal dose adjustment, 23.8% did not have an adjusted dose. According to the SmPC guidelines, a 50% dose reduction is necessary for the use of Fluconazole in patients with creatinine clearance lower than 50mL/min. 

For some rarely issued restricted antimicrobial drugs requiring renal dose adjustment (Levofloxacin, Ceftazidime/Avibactam, Cefpodoxime), none of the included requests had an adjusted dose. Consequently, a special caution is necessary in renal dose evaluation when dispensing these agents.

The results of this study showed a significant proportion of requests needing renal dose adjustment (14.2%) of which 39.1% did not have an adjusted dose. In this study, a clinical pharmacist monitored renal dose adjustment of restricted antimicrobial drugs and collaborated with other members of the A-team in recommending appropriate dosages. The A-team informed the prescriber about the dose correction of restricted antimicrobial drug. In a study published in 2021, clinical pharmacists provided interventions for antimicrobial therapy use, with dose adjustment being the most common intervention (42%). The three most common antibiotics requiring intervention were Meropenem, Piperacillin/Tazobactam and Vancomycin, showing similarities compared to the results obtained in our study [46]. Another study in which the prevalence of inappropriate dosing of antibiotics in patients with CKD was assessed also showed Piperacillin/Tazobactam, Meropenem and Vancomycin to be the most commonly prescribed antibiotics requiring dose adjustment [47]. A study that quantified the incidence of ADEs associated with broad-spectrum antibiotic use in hospitalized patients showed that 17.1% patients experienced ADEs. Piperacillin/Tazobactam, Meropenem, Doripenem, Vancomycin and Datpomycin were five most frequently prescribed antibiotics, with ADE occurrence observed in 20.7%, 16%, 15.4%, 19.6% and 11.8% of patients [24]. In our study, Doripenem and Daptomycin were not requested.

In order to ensure safe drug use and avoid ADRs, drug dose monitoring should be conducted. Hospitalized patients are more vulnerable to ADEs than outpatients. Non-adjusted renal drug doses can increase the risk of ADRs and compromise a patient’s treatment. The importance of the A-team in these endeavors should receive more attention. By supervising the use of antimicrobial therapy, the role of the A-team highlights the importance of controlling renal dose adjustment in AMS in order to ensure patient safety. Despite numerous recommendations, a large number of hospitals still do not provide an organized A-team. It should be highlighted that the implementation of the A-team improves the quality of provided healthcare and ensures patient safety [13].

Future strategies need to emphasize possible ADRs caused by inappropriate antimicrobial drug doses. These results contribute to the awareness of this problem and should be an indicator of future AMS strategies. ADRs can be misinterpreted, causing a prescribing cascade and prolonging hospitalization [48,49]. The collaboration is mandatory in planning AMS strategies. Further studies should be conducted in order to emphasize AMS interventions regarding patient safety. Research data on interventions facilitate and enhance the development of better recommendations for AMS. Hospitals should have an established system for reporting medication errors (MEs) and standard operating procedures. Hospitals should develop technical and documentation platforms for reporting MEs as part of AMS. The hospital’s standard operating procedures should include a developed platform that records MEs and provides feedback and a learning loop in order to improve care quality and patient safety. Our hospital has implemented a standard operating procedure and platform for ME reporting. Members of the working group for medication errors are authorized by the Commission for the quality of healthcare to carry out announced control of medication errors in prescribed pharmacotherapy, at least twice a year, by inspecting prescribed pharmacotherapy and discharge letters. The team conducting the control fills in the report on the control of pharmacotherapy. After processing the collected data, the working group prepares a report for the Commission at least twice a year on determined and/or potential medication errors, with a proposal on how to prevent the identified errors as well as the circumstances that can lead to errors. Medication errors observed in practice are used as a basis for mandatory education on the medication errors of healthcare workers.

With increasing rates of antimicrobial resistance and a small number of new antimicrobial drug arrivals on the pharmaceutical market, optimization of antimicrobial therapy is crucial. Results of AMS interventions should be published, emphasizing the quality of healthcare delivery in order to ensure patient safety. The World Health Organization (WHO) issued a global action plan on AMR, recommending that each country should develop and implement a national action plan [50,51]. The results of AMS intervention studies should be incorporated into national health action plans.

## 5. Limitations

Requests for restricted antimicrobial drugs in the Department for Cardiac and Transplantation Surgery were not included in the analysis since they are to be used in another work. Other aspects of the safe use of restricted antimicrobial drugs, such as drug–drug interactions or dose intervals, were not analyzed for the purposes of this study, even though they were included in the AMS practices.

## 6. Conclusions

Dose adjustment according to renal function is an important segment of antimicrobial stewardship. A significant proportion of requests requiring renal dose evaluation was incorrectly adjusted, which therefore highlights the importance of the A-team in restricted antimicrobial therapy optimization. Non-adjusted doses of restricted antimicrobial drugs can increase the possibility of ADRs and compromise pharmacotherapy outcomes and patient safety. Meropenem, Ciprofloxacin, Piperacillin/Tazobactam, Vancomycin, Colistin and the antimycotic Fluconazole were identified as restricted antimicrobial drugs that most often required dose evaluation and dose adjustment according to impaired renal function.

## Figures and Tables

**Table 1:** Patient characteristics.

Characteristic	N (%)
Number of patients	913
Male	540 (59.1)
Mean age, years	67.5
<65	323 (35.4)
65–75	259 (28.4)
75–85	240 (26.3)
>=85	91 (10.0)
Number of requests for restricted antimicrobial drugs (RAD)	2890
Mean number of requests for RAD per patient	3.17
Number of different RAD per patient (range)	1.6 (1–9)
Number of patients with 2 or more RAD simultaneously	133
eGFR stage (KDIGO classification)	
G1 Normal or high	1332 (46.1)
G2 Mildly decreased	708 (24.5)
G3a Mildly to moderate decreased	280 (9.7)
G3b Moderately to severely decreased	230 (8.0)
G4 Severely decreased	198 (6.9)
G5 Kidney failure	142 (4.9)
General principles of antimicrobial therapy, n (%)	
Empiric	1039 (33.0)
Directed	945 (30.0)
Prophylaxis	292 (9.3)
Continuation	874 (27.7)
Most frequently isolated microorganism, n (%)	
*Acinetobacter baummannii*	169 (17.9)
*Klebsiella pneumoniae*	140 (14.8)
*Pseudomonas aeruginosa*	128 (13.5)
*Escherichia coli*	87 (9.2)
*Clostridiodes difficile*	81 (8.6)
Number of requests per department, n (%)	
Department of gastroenterology, hepatology and clinical nutrition	284 (9.8)
Department of nephrology	244 (8.4)
Department of clinical immunology and rheumatology	232 (8.0)
Department of hematology	219 (7.6)
Intensive care unit, Department of internal medicine	198 (6.9)
Localization of infection, n (%)	
Respiratory tract	577 (20.0)
Urinary tract	524 (18.1)
Intra-abdominal	308 (10.7)
Sepsis	282 (9.8)
Skin and soft tissue	208 (7.2)

Abbreviations: RAD, restricted antimicrobial drug; eGFR, estimated glomerular filtration rate; KDIGO, Kidney Disease: Improving Global Outcomes.

**Table 2:** Number of requests for restricted antimicrobial drugs with and without dose adjustment.

Restricted Antimicrobial Drug	Total Number of Requests	Number of Requests Which Required Dose Adjustment according to Renal Function	Unadjusted Dose	Unadjusted Dose/Number of Requests That Required Renal Dose Adjustment (%)
Meropenem	540	125	54	43.2
Ciprofloxacin	455	121	33	27.3
Piperacillin/Tazobactam	195	39	10	25.6
Vancomycin	290	35	20	57.1
Colistin	113	24	16	66.7
Fluconazole	230	21	5	23.8
Cefepime	63	9	2	22.2
Teicoplanin	17	8	6	75
Imipenem/Cilastatin	16	8	0	0
Ceftazidime/Avibactam	39	6	6	100
Amikacin	49	6	4	66.7
Levofloxacin	38	3	3	100
Fosfomycin	28	2	1	50
Ampicillin/Sulbactam	66	2	0	0
Sulfamethoxazole-trimethoprim	29	2	0	0
Cefpodoxime	8	1	1	100
Cefixime	2	0	0	0
Ceftazidime	4	0	0	0
Ceftriaxone	430	0	0	0
Ertapenem	18	0	0	0
Total	2630	412	161	

**Table 3:** Number of requests of restricted antimicrobial drugs which do not require dose adjustment according to renal function.

Restricted Antimicrobial Drug	ATC Classification	Requests, n
Linezolid	J01X	95
Vancomycin (oral use)	J01X	90
Moxifloxacin	J01M	49
Posaconazole	J02A	9
Anidulafungin	J02A	7
Isavuconazole	J02A	5
Micafungin	J02A	1
Fidaxomicin	A07A	1
Tigecycline	J01A	1
Itraconazole	J02A	1
Voriconazole	J02A	1

## Data Availability

The data were used exclusively for the research conducted as part of this study and were kept confidential.
